# Chromosomal characterization of Amazonian freshwater stingrays with evidence for new karyomorphs and XX/XY sex chromosomes

**DOI:** 10.1590/1678-4685-GMB-2018-0229

**Published:** 2019-11-14

**Authors:** Francisco Carlos de Souza Valentim, Jorge Ivan Rebelo Porto, Eliana Feldberg

**Affiliations:** 1 Laboratory of Animal Genetics, National Institute of Amazonian Research, Manaus, AM, Brazil.

**Keywords:** Elasmobranchs, cytogenetics, chromosomal rearrangements, sex chromosomes, karyotypic diversity

## Abstract

Cytogenetic studies in the subfamily Potamotrygoninae have provided valuable insights into the understanding of the evolution and diversification of its species. In the present study, the chromosomal features of seven nominal potamotrygonin species are provided: *Plesiotrygon iwamae* (2n=74, FN=120), *Potamotrygon amazona* (2n=66, FN=107), *P. constellata* (2n=66, FN=110), *P. leopoldi* (2n=64, FN=102), *P. motoro* (2n=66, FN=106) from four different localities, and *P. orbignyi* (2n=66, FN=106), *P. scobina* (2n=66, FN=104), from Central Amazon. Additionally, we found a new karyomorph in *P*. *wallacei*. We considered the localization of Nucleolus Organizer Regions (NORs), as well as the pattern of constitutive heterochromatin, as species-specific characters. We found an XX/XY sex chromosome system in *P. orbignyi*, and we suggest that *P. scobina* and *P. amazona* also possess the same sex chromosome system. Overall, the chromosomal evolution in this group appears to have progressed towards a reduction in diploid number, with a concomitant increase in the number of bi-armed and nucleolar chromosomes.

## Introduction

The monophyly of the order Myliobatiformes, which includes the freshwater stingrays of the subfamily Potamotrygoninae, is well established (Carvalho *et al.*, 2016b; Nelson *et al.*, 2016). The subfamily Styracurinae, represented by the marine genus *Styracura* (*S. schmardae* and *S. pacifica*), is more closely related to the Neotropical freshwater stingrays, as confirmed by morphological (Lovejoy, 1996; Carvalho *et al.*, 2004; Aschliman *et al.*, 2012) and molecular phylogenies (Naylor *et al.*, 2012a; b; Bertozzi *et al.*, 2016; Last *et al.*, 2016). However, Marramà *et al.* (2018) do not recognize Styracurinae as a genuine member of Potamotrygonidae.

The subfamily Potamotrygoninae (35 species in *Paratrygon*, *Potamotrygon*, *Plesiotrygon,* and *Heliotrygon*) is considered monophyletic and represents today the unique group of elasmobranchs that evolved in freshwater habitats. Potamotrygonins are adapted entirely to freshwater environments, and are restricted to the continental waters of South America, where they inhabit the hydrographic basins that drain into the Atlantic Ocean and the Caribbean Sea with the exception of the São Francisco River basin and coastal drainage to the west and south of the Parnaíba River (Thorson *et al.*, 1983; Carvalho *et al.*, 2003; Rosa *et al.*, 2010). The lack of an adequate management plan, combined with the increasing harvesting and trade of these fish, not only as food but also for the ornamental fish market, as well as their biological characteristics, such as their reduced fecundity, slow growth, and late sexual maturation, make them extremely vulnerable to extinction (Araújo *et al.*, 2004; Charvet-Almeida *et al.*, 2005; Duncan *et al.*, 2010).

The increasing interest in the evolution of Potamotrygoninae has emphasized the need for a proper taxonomic classification and a well-supported phylogeny to better address the issues related to the evolutionary history of the group and the diversification of the stingrays in freshwater environments (Carvalho *et al.*, 2003; Lovejoy *et al.*, 2006; Toffoli *et al.*, 2008; Rosa *et al.*, 2010; Carvalho and Lovejoy, 2011; Cruz *et al.*, 2012; Fontenelle and Carvalho, 2016; Carvalho *et al.*, 2016b).

Indeed, taxonomic problems still exist, as many species present considerable variation in color patterns, and the presence of several mid patterns of dorsal pigmentation yields various errors and misidentifications. Even though, the number of potamotrygonins recently described is remarkable (Carvalho and Lovejoy, 2011; Carvalho *et al.*, 2011; Carvalho and Ragno, 2011; Loboda and Carvalho, 2013; Fontenelle *et al.*, 2014; Carvalho *et al.*, 2016a; b; Fontenelle and Carvalho, 2017).

The use of cytogenetic tools for studies concerning the number, structure, function, and origin of chromosomal variation, and the evolution of rays has been proposed since the 1960’s (reviewed in Valentim *et al.*, 2014). However, rays are one of the fish groups for which the fewest chromosomal data are available (Rocco *et al.*, 2005). Yet, it is already clear that its karyotypes vary considerably, with no modal chromosome number or any foremost karyotype formula (Stingo and Rocco, 2001; Rocco *et al.*, 2005).

To date, only six out of 35 species of Neotropical freshwater stingrays have been cytogenetically studied. However, the level of karyotypic variation detected showed distinct diploid numbers, karyotype formulas, and sex chromosomal systems, which represents an essential source of variation that can be used as markers for the characterization of evolutionary processes within different potamotrygonins (Valentim *et al.*, 2006; 2013; 2014; Cruz *et al.*, 2011; 2015; Aichino *et al.*, 2013).

The present study aimed to increment cytogenetic data on the Potamotrygoninae and improve our understanding of the chromosomal evolution and taxonomy of this critical and poorly studied Neotropical fish group.

## Material and Methods

**Figure 1:**
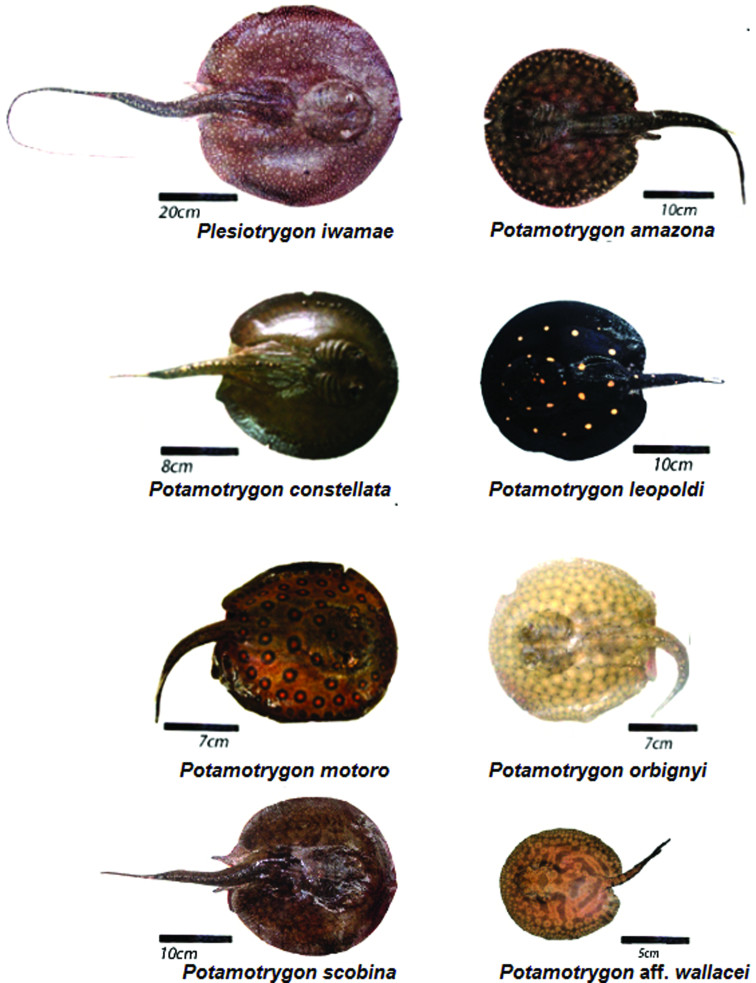
Stingray species analyzed in the present study.

We conducted cytogenetic analyses on *Plesiotrygon iwamae*, and on seven *Potamotrygon* species: *P. amazona*, *P. constellata*, *P. leopoldi*, *P. motoro*, *P. orbignyi*, *P. scobina*, and *P.* aff. *wallacei* collected in the Amazon region (Table 1, Figure 1). The collection of these individuals was authorized by the Information System of the Chico Mendes Institute for Biodiversity Conservation (ICMBio), an organ of the Brazilian federal government, through license number 28.095-1. The individuals were euthanized after clove oil anesthesia and deposited in the ichthyological collection of the National Institute of Amazonian Research (INPA) in Manaus (Table 1).

**Table 1:** List of the species and the number of individuals analyzed in the present study by sampling locations.

Species	Number of analyzed animals		Sampling locations	Coordinates	Vouchers number
	Female	Male			
*Plesiotrygon iwamae*	1	3	Janaucá/ Solimões River	3º17’41.57”S and 60º20’31.34”W	INPA-ICT 057993-057998
*Potamotrygon amazona*		1	Branco River/middle Negro River	1º23’45.98”S and 61º50’39.95”W	INPA-ICT 057981
*Potamotrygon constellata*	2	-	Janauacá/ Solimões River	3º17’41.57”S and 60º20’31.34”W	INPA-ICT 057985; INPA-ICT 057986
*Potamotrygon leopoldi*	4	3	Altamira/ Xingu River	3º20’29.69”S and 52º01’36.40”W	INPA-ICT 057987- 057992
*Potamotrygon motoro*	2	5	Catalão/ down Negro River	3º11’59.15”S and 59º53’59.03”W	INPA-ICT 057947-057953; INPA-ICT 057959-057962
*Potamotrygon motoro*	1	2	Janauacá/ Solimões River	3º22’03.66”S and 60º13’33.76”W	INPA-ICT 057963-057966
*Potamotrygon motoro*	2	1	Barcelos/ middle Negro River	0º27’05.39”S and 62º54’37.99”W	INPA-ICT 057954; INPA-ICT 057955
*Potamotrygon motoro*	-	4	Jauaperi/ middle Negro River	1º23’32.84”S and 61º38”56.43”W	INPA-ICT 057956- 057958
*Potamotrygon orbignyi*	1	-	Branco River/middle Negro River	1º23’45.98”S and 61º50’39.95”W	INPA-ICT 057967; INPA-ICT 057968
*Potamotrygon orbignyi*	-	1	Marchantaria/ Solimões River	3º11’9.80”S and 59º51’42.41”W	INPA-ICT 057969
*Potamotrygon orbignyi*	-	1	Altamira/ Xingu River	3º20’29.69”S and 52º01’36.40”W	INPA-ICT 057970
*Potamotrygon scobina*	-	3	Janauacá/ Solimões River	3º17’41.57”S and 60º20’31.34”W	INPA-ICT 057983-057985
*Potamotrygon* aff. *wallacei*	1	-	Itu/ middle Negro River	0º51’57.12”S and 62º46’24.63”W	INPA-ICT 057975

Mitosis was induced with biological yeast, according to Oliveira *et al.* (1988). Mitotic chromosomes were obtained using the air drying approach described by Bertollo *et al.* (1978) and adapted for stingrays by Valentim *et al.* (2006). The Nucleolus Organizer Region (NOR) was detected using silver nitrate (Ag-NOR), following Howell and Black (1980), while C banding for the detection of constitutive heterochromatin was conducted using barium hydroxide, as recommended by Sumner(1972). We arranged the karyotypes according to the size of chromosomes and sorted into metacentric (m), submetacentric (sm), subtelocentric (st), and acrocentric (a) types, based on the criteria proposed by Levan *et al.* (1964). To determine the fundamental number (FN) or the number of chromosome arms, we considered the metacentric, submetacentric, and subtelocentric chromosomes as having two arms, and the acrocentric chromosomes, a single arm.

## Results

**Figure 2:**
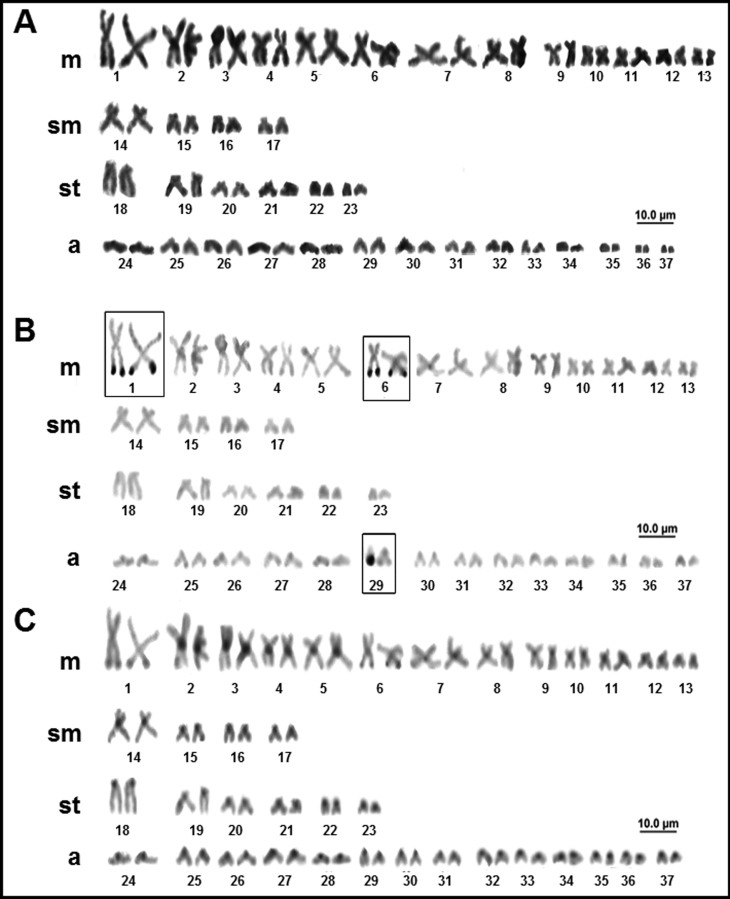
Karyotype of *Plesiotrygon iwamae*: (A) conventional Giemsa staining; (B) sequential AgNO_3_; (C) sequential C-banding.

*Plesiotrygon iwamae* (Rosa, Castello and Thorson 1987) has a diploid number of 74 chromosomes in both males and females, with a karyotype formula of 26m+8sm+12st+28a and FN = 120 (Figure 2A). Multiple NORs were detected in five sites over the terminal region of the long arms of pairs 1 and 6, and a single homolog of pair 29 (Figure 2B). Blocks of constitutive heterochromatin were detected in the centromeric region of most chromosomes, although some were relatively pale. In pairs 1 and 6, heterochromatic blocks were also observed in the terminal region of the long arms, coinciding with the NORs (Figure 2C).

**Figure 3:**
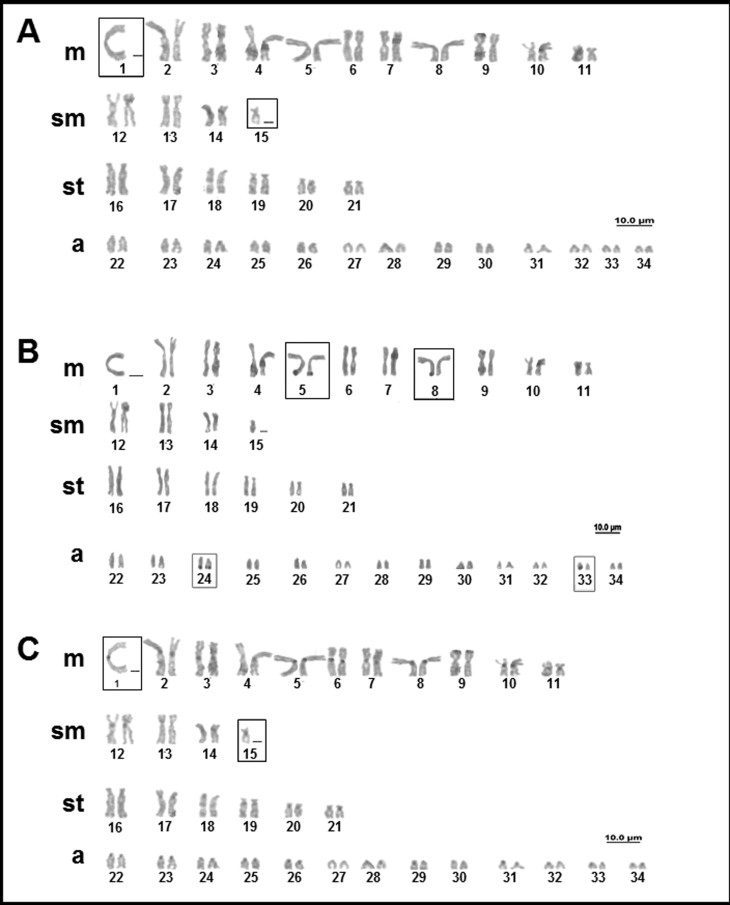
Karyotype of *Potamotrygon amazona* (male): (A) conventional Giemsa staining; (B) sequential AgNO_3_; (C) sequential C-banding.

For *Potamotrygon amazona* (Fontenelle and Carvalho 2017) only a single individual (male) was analyzed, and the diploid number was 66 chromosomes, with a karyotype formula of 21m+8sm+12st+25a and FN = 107 (Figure 3A). We detected the presence of two chromosomes with no homology, a large metacentric (pair 1) and a small acrocentric (pair 29). *A priori*, this variation could represent an XX/XY system of sex chromosomes, although this cannot be confirmed because there is no female karyotype confirmed yet for comparison. The individual presented multiple NORs located in the terminal region of the long arms of two chromosome pairs, corresponding to pairs 5 and 24, and one homologous of pair 8 (Figure 3B). Blocks of constitutive heterochromatin were detected in the centromeric region of most chromosomes, although some were relatively pale (Figure 3C).

**Figure 4:**
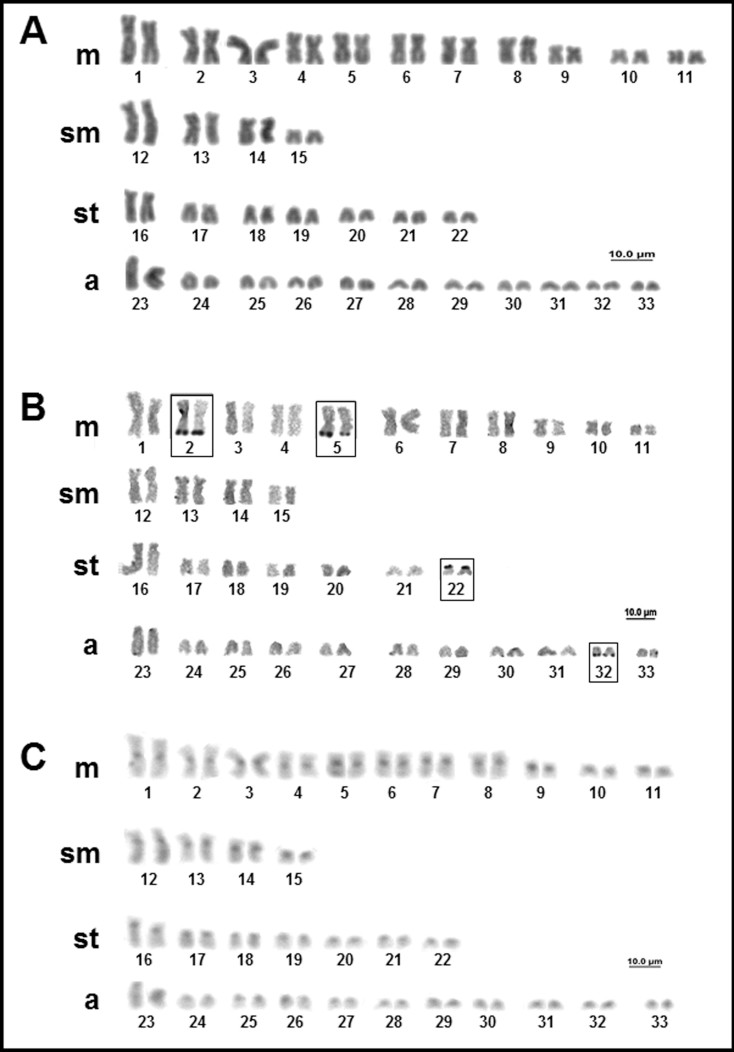
Karyotype of *Potamotrygon constellata* (female): (A) conventional Giemsa staining; (B) AgNO_3_; (C) C-banding.

For *Potamotrygon constellata* (Vaillant 1880) only females were analyzed, and the diploid number was 66 chromosomes, with a karyotype formula of 22m+8sm+14st+22a and FN = 110 (Figure 4A). The NOR is multiple, with up to six signals located in the terminal region of the long arms of pairs 2, 5, and 32, and the terminal region of the short arms of pair 22 (Figure 4B). Heterochromatin was found in the centromeric region of all the chromosomes (Figure 4C).

**Figure 5:**
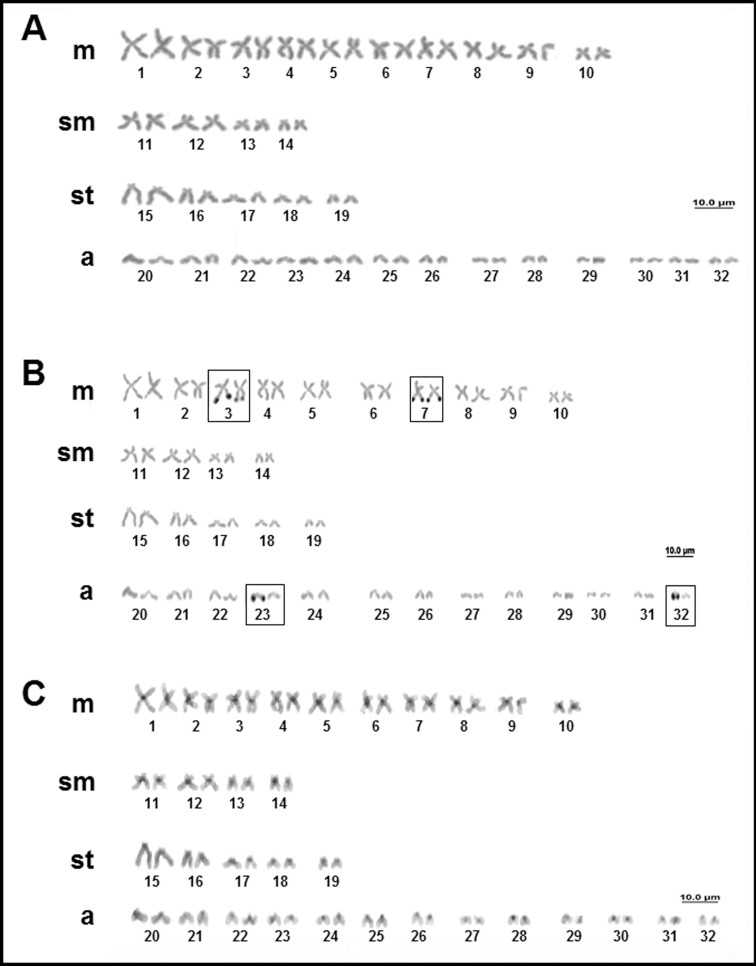
Karyotype of *Potamotrygon leopoldi*: (A) conventional Giemsa staining; (B) sequential AgNO_3_; (C) sequential C-banding.

*Potamotrygon leopoldi* (Castex and Castello 1970) has a diploid number of 64 chromosomes, in both males and females, with a karyotype formula of 24m+4sm+10st+26a, and a FN = 102 (Figure 5A). The NOR is multiple, with as many as six signals in the long arms of pairs 3 and 7, and in one homologous of pairs 23 and 32 (Figure 5B). We observed the constitutive heterochromatin in the centromeric region of all the chromosomes (Figure 5C).

**Figure 6:**
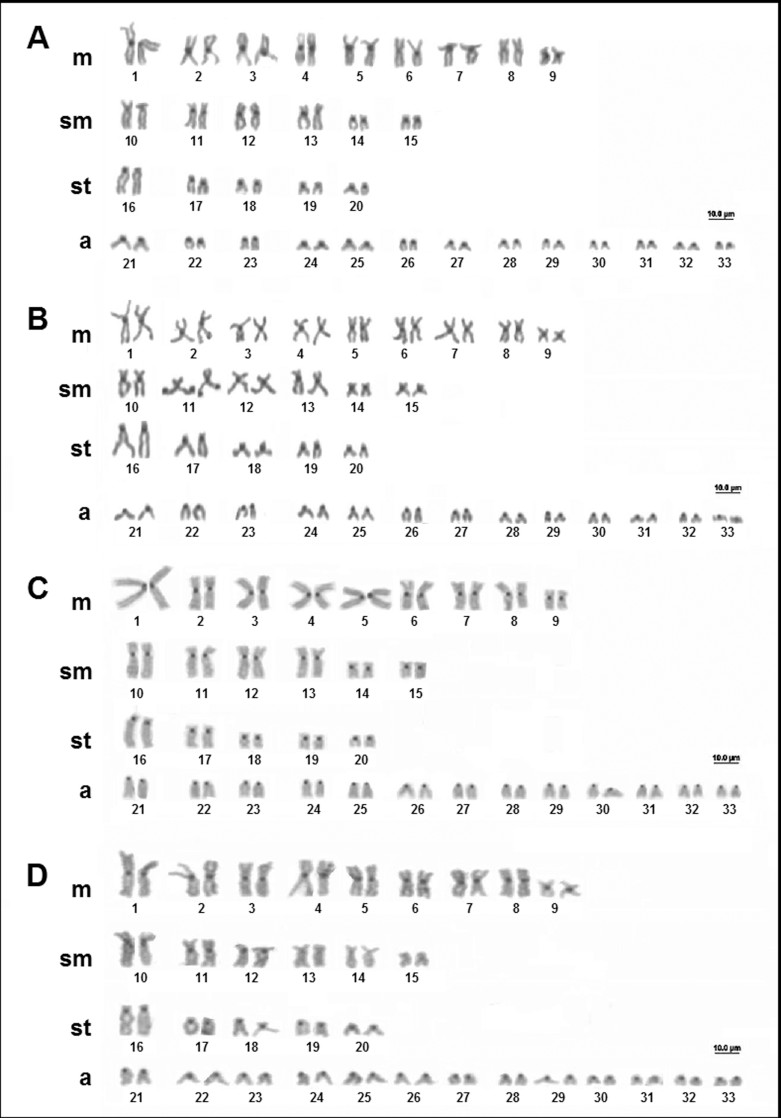
Karyotypes of *Potamotrygon motoro*: C-banding of individuals from different localities: (A) middle Negro River, Barcelos region; (B) Jauaperi River, middle Negro basin; (C) Catalão Lake, lower Negro basin; (D) Janauacá Lake, Solimões basin.

*Potamotrygon motoro* (Müller and Henle 1841) is the most widely distributed species of freshwater stingray. In the present study, individuals from four different localities in the Amazon basin were analyzed, including three from the middle Negro River and one from the Solimões River (upper Amazon) near the city of Manaus (Table 1). All the individuals showed a diploid number of 66 chromosomes, with a karyotype formula of 18m+12sm+10st+26a and FN = 106, with no differentiation of males and females (Valentim *et al.*, 2006). Constitutive heterochromatin was observed in the centromeric region of all the chromosomes, with no variation among localities (Figure 6), except for the sample from the Jauaperi River (a tributary of Negro River), which presented additional heterochromatic blocks in the terminal region of the long arms of pair 11 (Figure 6B).

**Figure 7:**
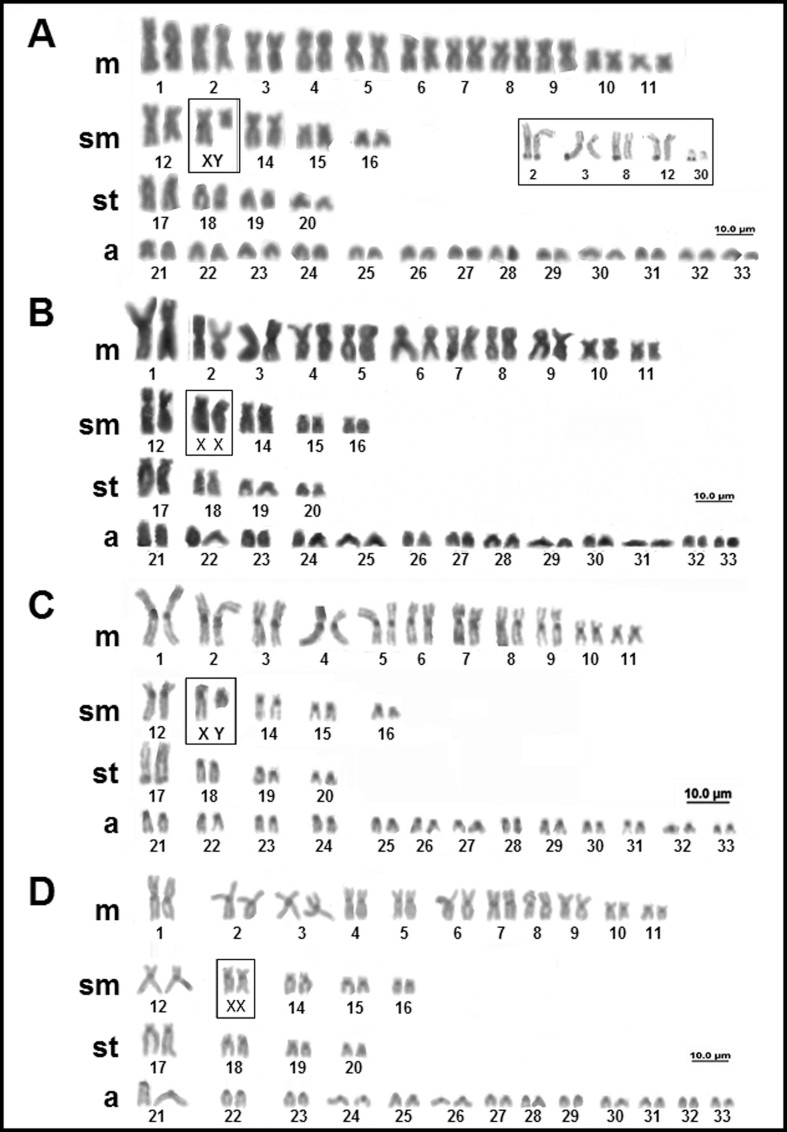
Karyotypes of *Potamotrygon orbignyi*: (A) Giemsa staining (male); (B) Giemsa staining (female); (C) C banding (male); (D) C-banding (female); AgNO_3_ inbox.

In *Potamotrygon orbignyi* (Castelnau 1855) all males and females individuals showed a diploid number of 66 chromosomes with a karyotype formula of 22m+10sm+8st+26a, and FN = 106. However, an XX/XY system of sex chromosomes is present (Figure 7A, B). The NOR is multiple, with as many as eight signals in the long arms of pair 2 and one homologous of pairs 3, 8, 12, and 30 (Figure 7, inbox). Constitutive heterochromatin was observed in the centromeric region of all the chromosomes (Figure 7 C, D).

**Figure 8:**
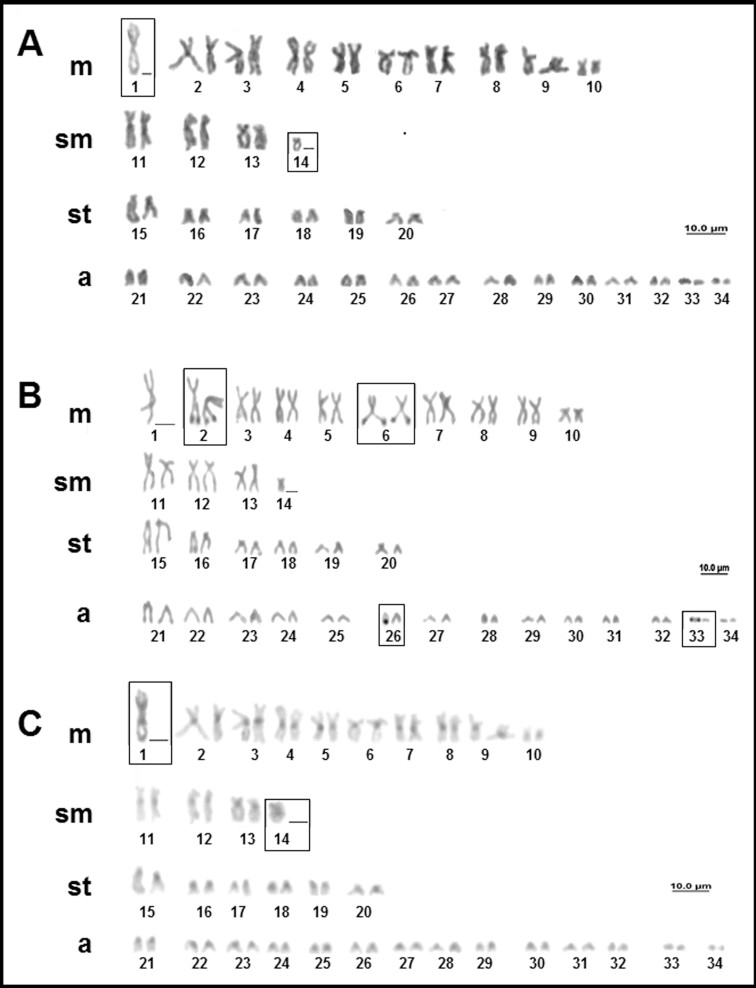
Karyotype of *Potamotrygon scobina*: (A) conventional Giemsa staining; (B) AgNO_3_; (C) sequential C-banding.

For *Potamotrygon scobina* (Garman 1913) only males were analyzed, and they had a diploid number of 66 chromosomes with a karyotype formula of 21m+7sm+12st+28a and FN = 108 (Figure 8A). As in the case of *P. amazona* we detected the presence of two chromosomes with no homology, a large metacentric (pair 1) and a small submetacentric (pair 14). *A priori*, this variation could represent an XX/XY system of sex chromosomes, although this cannot be confirmed because there are no female karyotypes available yet for comparison. All the individuals presented multiple NORs located in the terminal region of the long arms of two chromosome pairs, corresponding to pairs 2 and 6, and one homologous of pairs 26 and 33 (Figure 8B). Blocks of constitutive heterochromatin were detected in the centromeric region of most chromosomes, although some were relatively pale. The chromosome pair 1, which lacks a homologous, had a larger block of heterochromatin in the region of the centromere, which extends to the proximal regions in both arms (Figure 8C).

**Figure 9:**
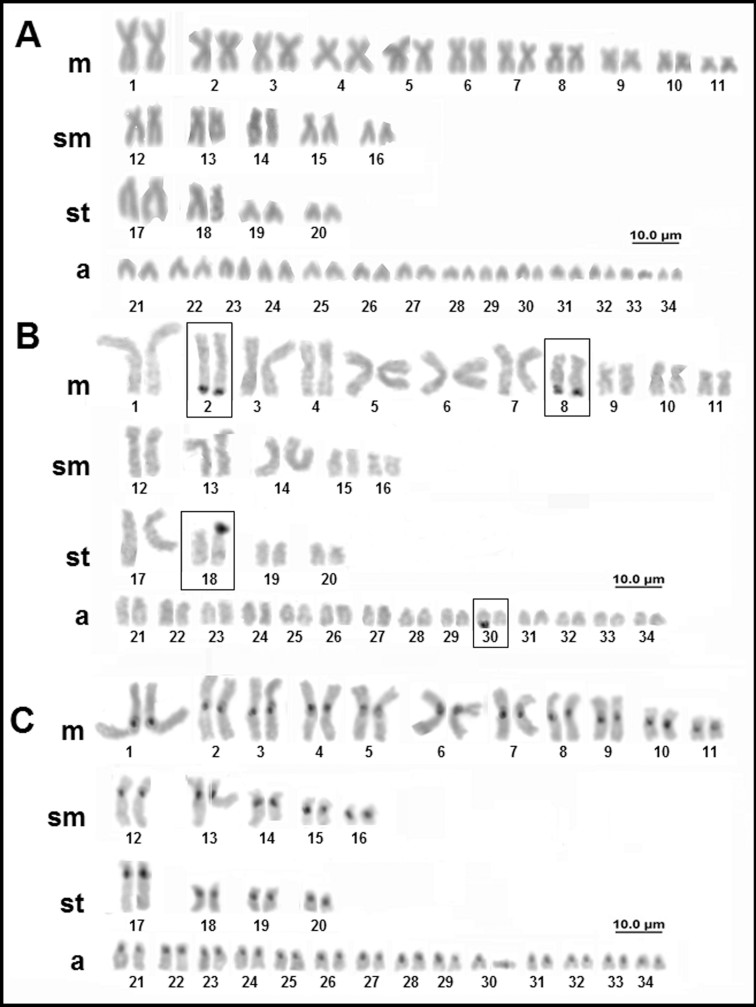
Karyotype of *Potamotrygon* aff. *wallacei*: (A) conventional Giemsa staining; (B) AgNO_3_; (C) C-banding.

For *Potamotrygon* aff. *wallacei* (Carvalho, Rosa, Araújo 2016), one individual (female) that was morphologically similar to *Potamotrygon wallacei*, presented a diploid number of 68 chromosomes, but with a different karyotype formula (22m+10sm+8st+28a and FN = 108) of those previously reported by Valentim *et al.* (2013) (Figure 9A). The NOR is multiple, with up to six signals located in the terminal region of the long arms of pairs 2 and 8, and one of the homologous of pair 30, as well as the short arm of one of the homologs of pair 18 (Figure 9B). Constitutive heterochromatin is in the centromeric region of all the chromosomes (Figure 9C).

## DISCUSSION

Only few karyological studies have been performed so far on potamotrygonins. Our study now increases this number from six to 15 cytogenetically studied species/morphospecies, as shown in Table 2. Except for the genus *Heliotrygon*, we have thus considerably advanced the knowledge on freshwater stingrays karyotypes, noting that they have different diploid numbers (varying from 2n=66-90) and very different karyotype formulas (from FN=104-120).

**Table 2:** Compilation of the karyotype data available for the freshwater stingrays and the data obtained in the present study (2n = diploid number; FN: fundamental number; NOR: Nucleolus Organizer Region; T: terminal; p: short arm; q: long arm; m: metacentric; sm: submetacentric; st: subtelocentric; a: acrocentric).

Species	Karyotype formulae	2n	FN	NORs Number	NORs pairs	NORs position	Sexual system	References
*Paratrygon aiereba*	4m+2sm+10st+74a	90Male/Female	106	2 a 4	-	T/pq	-	Valentim *et al*., 2006
*Plesiotrygon iwamae*	26m+8sm+12st+28a	74Male/Female	120	5 a 6	1, 6, 29	T/q	-	Present work
*Potamotrygon amazona*	21m+8sm+12st+25a	66Male	107	5 a 6	5, 8, 24	T/q	XY(?)	Present work
*Potamotrygon constellata*	22m+8sm+14st+22a	66Female	110	6	2, 5, 22	T/pq	-	Present work
*Potamotrygon falkneri -* upper Paraná River	20m+9sm+14st+22a/ 20m+10sm+14st+22a	65Male/66Female	108/110	10	-	T/q	X_1_X_1_X_2_X_2_/X_1_X_2_Y	Cruz *et al*., 2011
*Potamotrygon falkneri* - upper Paraná River	20m+9sm+18st+18a/ 20m+10sm+18st+18a	65Male/66Female	112/114	8	-	T/q	X_1_X_1_X_2_X_2_/X_1_X_2_Y	Cruz *et al*., 2011
*Potamotrygon leopoldi*	24m+4sm+10st+26a	64Male/Female	102	6 a 8	3, 7, 23, 32	T/q	-	Present work
*Potamotrygon motoro*	18m+12sm+10st+26a	66Male/Female	106	8 a 10	3, 5, 14, 20, 24	T/q	-	Valentim *et al*., 2006
*Potamotrygon* aff*. motoro* - upper Paraná River	21m+9sm+19st+16a 22m+8sm+20st+16a	65Male/66Female	114/116	8	-	T/pq	X_1_X_1_X_2_X_2_/X_1_X_2_Y	Cruz *et al*., 2011
*Potamotrygon* aff*. motoro -* upper Paraná River	20m+10sm+25st+10a/ 22m+10sm+26st+10a	65Male/66Female	120/122	10	-	T/pq	X_1_X_1_X_2_X_2_/X_1_X_2_Y	Cruz *et al*., 2011
*Potamotrygon motoro* Paraná River - Corrientes, Argentina	13m+15sm+23st+14a/ 14m+16sm+22st+14a	65Male/66Female	116/118	4 a 6	-	T/pq	X_1_X_1_X_2_X_2_/X_1_X_2_Y	Aichino *et al*., 2013
*Potamotrygon orbignyi*	22m+10sm+8st+26a	66Male/Female	106	8 a 10	2, 3, 8, 12, 30	T/q	XX/XY	Present work
*Potamotrygon scobina*	19m+7sm+12st+28a	66Male	104	6 a 8	2, 6, 26, 33	T/q	XY(?)	Present work
*Potamotrygon wallacei*	19m+8sm+10st+30a/ 20m+8sm+10st+30a	67Male/68Female	104/106	6 a 9/6 a 10	X, 8, 18 23, 30/XX, 8, 18, 23, 30	T/q	XX/X0	Valentim *et al*., 2013
*Potamotrygon* aff. *wallacei*	22m+10sm+8st+28a	68Female	108	6 a 8	2, 8, 18, 30	T/q	-	Present work

Morphological and molecular phylogenies recover two clades in the Potamotrygoninae: *Paratrygon* + *Heliotrygon* and *Potamotrygon* + *Plesiotrygon* (Carvalho and Lovejoy, 2011). If we superpose the chromosomal data over the phylogenies, it is possible to observe that the reduction in diploid number correlates with a decrease in the number of acrocentric chromosomes. For instance, *Paratrygon aiereba* (2n = 90; 4m+2sm+10st+74a) possesses the highest amount of chromosomes, mostly acrocentric, among potamotrygonins (Valentim *et al.*, 2006). Since we still do not know the karyotypes of *Heliotrygon* species, comparisons in this clade are not possible. In the other clade, *Plesiotrygon iwamae* (2n = 74; 26m+8sm+12st+28a) is the only cytogenetically analyzed species of the genus (*P. nana* remains to be karyotyped). It possesses a higher diploid number than the genus *Potamotrygon*, its sister group. Apparently, the potamotrygonins have undergone a reduction in diploid number (2n = 90 => 2n = 74 => 2n = 64–68) along their evolutionary history.

The fundamental chromosome numbers of these different taxa are highly consistent with Robertsonian chromosomal rearrangements that could have occurred during evolution. In the clade formed by *Plesiotrygon iwamae* (FN = 120) and *Potamotrygon* species (FN = 101–122), the differentiation of the karyotypes is based on centric fusions, in addition to possible pericentric inversions. The comparison of the two clades (*Paratrygon* and *Potamotrygon* + *Plesiotrygon*) reveals that, while there is a significant reduction in chromosome number, the number of arms varies, but not as drastically.

Overall, the available chromosomal data for the genus *Potamotrygon* indicate that, based on their diploid numbers, the species are organized in three groups, i.e., 2n = 64, 66, and 68 chromosomes. There are internal deviations, however, associated with heteromorphism of the sex chromosomes (Cruz *et al.*, 2011; Valentim *et al.*, 2013), although the analysis of this arrangement is still preliminary, given that only 15 of the 30 valid *Potamotrygon* species have been karyotyped to date. Individuals allocated to the same species have been distinguished by the analysis of karyotypes, as in the cases of *Potamotrygon wallacei* and *Potamotrygon* aff. *wallacei*, and *P. motoro* and *P.* aff. *motoro* (Table 2).

A remarkable feature in the cytotaxonomic study of fish is the presence of heteromorphic sex chromosomes in males and females, in contrast to the normal homomorphic chromosomes present in the vast majority of species (Kitano and Peichel, 2012). Heteromorphic sex chromosomes have been described in few marine and freshwater stingrays (for reference see Valentim *et al.*, 2014). In Potamotrygoninae, the sex chromosome is an apomorphic trait detected only in *Potamotrygon*, in which three different sex-determination systems have been observed: XX/XY, XX/X0, and X_1_X_1_X_2_X_2_/X_1_X_2_Y (Valentin *et al.*, 2014). The first was found in *P. orbignyi* and possibly also *P. scobina* and *P. amazona* (present study). The second, in *Potamotrygon wallacei* (Valentim *et al.*, 2013). The third, in *P.* aff. *motoro* and *P. falkneri* (Cruz *et al.*, 2011) and in *P. motoro* (Aichino *et al.*, 2013). Presently, the simple sex-determination systems were found in rays from the Amazon basin (Valentim *et al.*, 2006; 2013; present study), whereas the multiple sex-determination system was found in rays from the Paraná basin (Cruz *et al.*, 2011; Aichino *et al.*, 2013).

Despite the advances in the cytogenetics of freshwater stingrays and comparisons with data available for the Rajiformes and Myliobatiformes (Valentim *et al.*, 2014), the direction of chromosomal evolution in Potamotrygonidae still remains unclear, given the lack of chromosome data from the *Styracura* species. Available chromosomal data indicates a progressive reduction in the number of chromosomes, given that most representatives of other myliobatiform families have low diploid numbers.

In both freshwater and marine Batoidea, the constitutive heterochromatin was invariably located in the centromeric region of all the chromosomes, and the NORs were multiple (Stingo *et al.*, 1995; Rocco *et al.*, 2002; 2005; 2007; Valentim *et al.*, 2006; 2013; Cruz *et al.*, 2011; Aichino *et al.*, 2013). This pattern was also found in the present study, although individuals of *P. motoro* from one locality (Jauaperi River) presented terminal heterochromatic blocks on the long arms of a single chromosome pair.

It is interesting to note that differentiated heterochromatin segments were not found in the probable sex chromosomes of *P. amazona*, *P. orbignyi*, and *P. scobina*, even though such segments are found in many other species of Neotropical fish (Born and Bertollo, 2000; Almeida-Toledo and Foresti, 2001; Terencio *et al.*, 2013). Cruz *et al.* (2011) and Aichino *et al.* (2013) recorded a similar lack of differentiation in the multiple sex chromosomes of the *Potamotrygon* species from the Paraná basin. Overall, a small amount of heterochromatin with an essentially centromeric distribution appears to be a plesiomorphic trait in the stingrays.

All the freshwater stingray species analyzed up to now present multiple NORs located in the terminal region of the long arms. However, *Potamotrygon constellata* and *Potamotrygon* aff. *wallacei* presented one chromosome pair, in which the NOR is located on the short arms. The range of active NORs (Ag-NORs) varied, from four sites in *Paratrygon aiereba* (Valentim *et al.*, 2006), six sites in *Plesiotrygon iwamae*, and up to eight sites in the *Potamotrygon* species.

The karyotype diversity found in the Potamotrygoninae indicates that the occurrence of chromosomal rearrangements, both Robertsonian and non-Robertsonian, is strictly associated with the karyotypic evolution of the group. These rearrangements would also account for the variability in the NORs and the presence of sex determination mechanisms, given the sex chromosomes were not differentiated through heterochromatinization, but rather by rearrangements. The simple and multiple sex chromosome systems are derived traits in the chromosomal evolution of this group, and were only found in *Potamotrygon.* Therefore, all these features qualify the freshwater stingrays, especially *Potamotrygon*, as a promising model to investigate the evolution of sex chromosomes and chromosomal evolution in general.
